# Science in the clinic: a qualitative study of the positioning of MD-PhDs in the everyday clinical setting

**DOI:** 10.1186/s12909-018-1222-2

**Published:** 2018-05-25

**Authors:** Pernille Andreassen, Mette Krogh Christensen

**Affiliations:** Centre for Health Sciences Education, Aarhus University, INCUBA Science Park – Skejby, Palle Juul-Jensens Boulevard 82, building B, 8200 Aarhus N, Denmark

## Abstract

**Background:**

MD-PhDs have been hailed as significant to the advancement of medicine and health care. Yet when it comes to which positions MD-PhDs should be holding in the clinic and the academic world, there seems to be no real consensus. This article examines the ways in which a PhD-degree may contribute to medical doctors’ professional practice in the clinic and discusses the positioning of MD-PhDs in the clinic.

**Methods:**

The study is explorative and qualitative, based on interviews with MD-PhDs, their physician colleagues without a PhD-degree, and their leaders. Positioning theory was applied as the analytical framework for data analysis.

**Results:**

We found two opposing positions cutting across the groups of informants with one side critiquing the MD-PhDs for not doing enough research and for using the PhD-degree to climb the career ladder, while the other side emphasized the ways in which MD-PhDs increase the clinical focus on evidence-based medicine and integrate it with clinical decision making, thereby enhancing patient care.

**Conclusions:**

A debate is needed to establish more clearly how we wish to position MD-PhDs in the clinic, which in turn will give us a better idea of how many to educate and how to make better use of their competencies.

## Background

In the world of biomedical research, MD-PhDs and physician-scientists have been hailed as key contributors to biomedical advances and the improvement of health care [1–3]. However, when it comes to which positions MD-PhDs should be holding in the clinic and the academic world, there seems to be no real consensus, which is the subject we will be studying in this article.

The MD-PhD-degree can be described as a dual doctoral degree that includes a degree in medicine (MD) and a doctorate of philosophy (PhD), which is a scientific research education resulting in an advanced post-graduate degree, usually based on 3 years of study and a dissertation. As such MD-PhDs are prepared for careers as physician-scientists. In the US, MD-PhDs typically spend most of their time conducting research and dividing their remaining time between clinical service, teaching, and administrative activities [4]. However, the range of the professional options of MD-PhDs is broad and varies internationally [4, 5].

In many biomedically advanced countries, such as USA, Canada, Japan, and Germany, concern has long been growing over a lack of physician-scientists in general and MD-PhDs in particular [6–9]. As early as in the 1970s, warnings were issued that physician scientists were becoming “*an endangered species*” [10] (p. 1254). Currently, some argue that the continuing decrease in the number of MD-PhDs poses a threat to the advancement of biomedical science and the translation of research findings to clinical practice [11–13].

The present empirical study takes place in a Danish context which differs from the described international situation in significant ways. First of all, the number of MD-PhDs has been increasing in Denmark since 2006 where a political agreement called *Globaliseringsaftalen* (literally *The Globalization Agreement*) resolved to boost investment in science and research in a wide sense, including earmarking resources for increasing the overall intake of PhD-students at Danish universities. Accordingly, Danish universities enroll approximately 60% more PhD students today than in 2006, including MD-PhDs [14]. Hence, while the international debate has concerned itself with the possible causes of and resolutions to the decrease in MD-PhDs and physician-scientists, the discussion in both the professional medical press and the daily press in Denmark has revolved around whether too many MD-PhDs are being produced, and - since the PhD-degree is publically funded in Denmark - whether the MD-PhDs are worth the investment. For example, in 2014 approximately 320 medical students graduated from Aarhus University (Denmark’s second largest university). The same year 77 MD-PhDs graduated from Aarhus University [15]. Thus MD-PhDs constituted approximately 20% of all MDs from that year. This is a higher percentage than is seen in countries such as the USA or European countries such as Finland, Norway, and Germany [16, 17]. In particular, critics of the increased number of MD-PhDs have focused on the limited research activity in MD-PhDs [18] and have speculated that some physicians merely do a PhD as a “shortcut” to being admitted to the clinical specialization of their choice and then subsequently stop doing research altogether. Advocates for the increase in the number of MD-PhDs in their turn have argued that MD-PhDs contribute in others ways than exclusively doing research [19, 20]. Another significant distinction between the Danish and the international situation is that Danish MD-PhDs tend to go back to work more or less full time in the clinic, while only a minority continue on doing primarily or fulltime research [21].

To the best of our knowledge no empirical studies have been made concerning the ways in which a PhD-degree may contribute to MD-PhDs’ professional practice in the everyday clinical setting. Hence in this study, we have explored how MD-PhDs are positioned in the clinic, i.e. which competencies, rights, and duties they are attributed. In particular, we were interested in knowing how different relevant staff members position MD-PhDs in the clinic, and the study is therefore based on interviews with not only MD-PhDs, but also physicians without a PhD-degree (i.e. colleagues), and leaders.

## Methods

This study used a qualitative and explorative approach [22] to gain access to the views, opinions, and experiences of MD-PhDs, their (physician) colleagues and their leaders.

### Data collection

Data collection was carried out by the first author and took place from March through June 2016 and was primarily carried out in Central Denmark Region and to a lesser extent in North Denmark Region.

In order to get a wide variety of perspectives on the role of MD-PhDs in the clinic and thereby strengthening the validity of the study, we wanted to include different types of hospitals (university and regional), different kinds of doctors (medical and surgical) as well as including MD-PhDs, their colleagues and leaders (see Table 1 for an overview). We assumed there would be a difference between being an MD-PhD in medical and surgical specialties and chose one of each, based on the specialties being represented in both regional and university hospitals.

**Table 1:** Overview of informants and interviews

Individual interviews							Group interviews
Participants	MD-PhDs (7 male, 7 female)		Colleagues of MD-PhDs (physicians without PhD) (5 male, 2 female)		Leaders (Executive consultants) (5 male, 1 female)		Leaders (Appointment committees) (22 male/14 female)
	Medical specialty	Surgical specialty	Medical specialty	Surgical specialty	Medical specialty	Surgical specialty	
University hospital	5	2	1	1	1	1	Four group interviews; 36 physicians interviewed in total. Participants made up four different appointments committees, covering four different specialties (three surgical and one medical).
Regional hospital 1	4	1	4	1	1	1	
Regional hospital 2	1	–	–	–	1	–	
Regional hospital 3	–	1	–	–	–	1	

Recruitment was carried out in different ways. First six executive consultants from selected wards were contacted via e-mail and then by phone. They all agreed to participate in interviews which then took place at their office. After the interview had taken place, the executive consultants sent out an email to the physicians in their ward, encouraging them to participate in an interview. After a week the executive consultants were encouraged to send out a reminder to the physicians, which they kindly did, and this resulted in a four more physicians volunteering. The physicians who responded were contacted individually via e-mail and were informed that participation was voluntary, and that they were under no obligation to participate, if they did not want to, and that their leader would not be informed whether they participated or not. In total 14 MD-PhDs and 7 (physician) colleagues agreed to participate in interviews.

Furthermore, five appointment committees were selected based on being representative of both medical and surgical specialties and being different in terms of size and research tradition (some being more research oriented than others) as we thought these criteria might influence the perception of MD-PhDs. The coordinators of the committees were contacted via e-mail and passed on our interview request to the committees. One coordinator never responded despite e-mails and phone calls, but the other coordinators arranged for the first author to meet with the remaining four appointment committees for half an hour either before or after they carried out job interviews for residency training. Participants in the appointment committee group interviews were senior hospital physicians, clinical associate professors, head consultants, specialist consultants, Heads of Degree Program and junior doctors from hospitals throughout the Central Denmark Region and the North Denmark Region.

All participants of this study contented to participate verbally and were informed that they could withdraw from the study at any time. They were provided with the first author’s e-mail address and phone number in case they had questions or wanted to withdraw from the study. Two of the participants wished to be informed if they were quoted in the final article; they were contacted and approved of the quotes included in the article.

#### Interviews

The interviews were semi-structured and based on an interview guide [23, 24]. The guide included a range of themes and open-ended questions pertaining to being/having an MD-PhD in the clinic, such as expectations of MD-PhDs, experiences with MD-PhDs and the role of science in the clinic.

Individual interviews lasted between 45 min and 1½ hours, while the group interviews lasted between 30 and 40 min. All interviews were audio-recorded and were carried out by the first author who is an experienced ethnographer and interviewer. All interviews took place in hospital offices where the interviewees could speak freely, which they were invited to do, after the interviewer had assured them that all data would be treated anonoumously, and all personally identifiable information left out. The interviewees all seemed comfortable talking about the subject of MD-PhDs; some voicing strong opinions.

### Ethical considerations

The study was approved by the Danish Data Protection Agency (J. No. 2015-57-0002, Sequential No. 211). Approval by The Central Denmark Regional Committees on Biomedical Research, under which this study is classified, was not required, because our study did not involve medical products, experiments including patients, etc.

The American Anthropological Association’s code of ethics was followed [25]. In particular we have chosen not to specify which hospitals, wards, and medical specialties we have included in the study in order for all informants to stay anonymous and be able to speak freely. All informants were informed that data would be treated confidentially and anonymously before interviews commenced.

### Analysis

Transcriptions of the interviews were carried out by the first author and an accomplished secretary. The transcriptions were read through several times by the first author and subsequently analyzed following the qualitative content analysis approach described by Emerson et al. [26]. First, initial codes were generated, and initial memos were written and discussed by the authors. By investigating relations between codes, overall themes and patterns were selected and explored in relation to the full data set. Themes and codes were discussed among the authors before the final analysis was conducted.

The excerpts used in this article were translated from Danish to English by the first author and reviewed by the second author. We have chosen to include a fairly large number of interview excerpts as a form of “*textual evidence*” [27] (p. 48) in order to strengthen the transparency and thereby the validity of the analysis. Furthermore, by including informants from different types of hospitals and different specialties (medical and surgical) as well as including MD-PhDs, their colleagues and leaders, we have made use of what Patton terms “*triangulation of sources*” [28] (p. 1193) as a form of validity check. Finally, we have taken great care in the interpretation of data to include “*different real world nuances*” [28] by including informants’ different viewpoints.

#### Positioning theory as analytical framework

In this article, we use positioning theory as our overall analytical framework. Positioning theory is a social constructivist approach which allows us to highlight how multiple actors (i.e. MD-PhDs, colleagues, and leaders) position themselves, position others and are themselves positioned, emphasizing different types of rights and duties attached to the PhD-degree.

Positioning theory seeks to break with theories of social behavior that regard rules and roles as static and stable and social encounters as formal and ritualistic. Positioning theory focuses instead on the dynamic and changeable character of social encounters, highlighting the variable positions people assign to themselves and to others in unfolding social situations [29, 30].

The positioning of oneself and others is seen to be revealed discursively, i.e. it is through talk and conversation that individuals engaged in talking negotiate positions for themselves and others. In this sense, discourses make available positions for individuals in terms of the discursive material available in the local moral order within which people allocate a variety of positions to themselves and each other [31]. Below we describe the three interrelated components of positioning theory which constitutes the positioning triangle, consisting of *Positions*, *Acts* and *Storylines* [32], which we will use to describe the positions adopted and ascribed to the MD-PhD-position by our informants.

##### Positions

Positions are described as momentary “*clusters of rights and duties with respect to what can legitimately be said and done by whom*” [33] (p. 186). Rights are defined as anticipatory or retrospective justifications for the propriety of demands for actions by someone else, i.e. something you can claim or expect from others, while duties refers to the acts that one is expected to perform, i.e. what others expect you to do [34]. Thus the content of a position derives from (disputable) local, moral standards with respect to the rights and duties one is enabled to carry out as an occupant of a position. Someone can accept a position, but may also be forced to defend a position, renounce a position, or contest their positioning by others by drawing on alternative discursive resources and systems of meanings to construct new positions for themselves or others. In this article, we will touch on three modes of positioning: Positioning oneself, positioning others, and being positioned by others.

##### Acts

An act is defined as “*a socially meaningful and significant performance*” [32] (p. 6). Acts are social actions which have been interpreted in a socially meaningful way, and positions are negotiable exactly because acts make it possible to reinterpret positions. Importantly, the same acts can be interpreted in different ways, because a position (rights and duties) can be challenged and called into question, and reversely being positioned in a particular way affects which acts one can meaningfully opt for and make use of.

##### Story lines

Storylines have to do with what can be expected in specific social episodes, providing an order, convention or (moral) framework for what one can, might and ought to do [32], thereby making actions meaningful. Storylines may unfold according to socially accepted conventions. However, any encounter might develop among more than one story-line and support several story-lines developing at the same time.

##### Footing

Finally, we will be referring to the concept of *footing*, a concept which positioning theory borrows from Goffman [35]. “Having a footing” refers to the manner in which one can enter into a conversation or social situation unchallenged. Someone with “footing” has recognized rights and is consequently someone you listen to and whose opinion matters and is taken notice of [30].

## Results

In the following we will present participants’ (i.e. MD-PhDs, colleagues and leaders) storylines regarding MD-PhDs and their positions in the clinic (see Table 2 for an overview). It is important to emphasize that there was not consensus either within the participant groups or between the groups as to the positioning of MD-PhDs.

**Table 2:** Informants’ storylines regarding the PhD-degree and the positioning of MD-PhDs in the clinic

MD-PhDs	The PhD-degree is academic capital
	The PhD-degree is a way to improve evidence-based clinical practice
	The PhD-degree entails a duty to do research
Colleagues (physicians without a PhD-degree)	MD-PhDs are resource persons
	MD-PhD hold an unjustly favored position
Leaders	MD-PhDs are resource persons
	The PhD-degree entails a duty to do research
	MD-PhD hold an unjustly favored position

### MD-PhDs’ storylines

#### The PhD-degree is academic capital

All of the MD-PhDs who were interviewed considered the PhD-degree as a way to improve their career options. However, there was great variation as to why the MD-PhDs had done a PhD in the first place. While some of the MD-PhDs conveyed great passion for and interest in doing research, others mainly regarded the degree as a career opportunity. To most, however, doing a PhD-degree was mix of both reasons. A typical answer to the question as to why one had done a PhD came from a staff doctor:

*There were two reasons really. First of all because I think it is part* [of being a physician], *being able to see through research results, and I’m also interested in it* [research]. *And on the other hand it’s probably also a little bit of CV speculation. If I want to be* [a certain kind of medical specialist], *I knew there might be a battle for it, and.. so there was definitely also an ulterior CV motive*.

MD-PhD, medical specialty, regional hospital

A prominent storyline in the Danish medical educational system is that you need a PhD to be admitted to a clinical specialization at a university hospital or at one of the popular medical specialties, and as shown in the above quote, some of the MD-PhDs portrayed the PhD degree as a way of positioning themselves in terms of achieving a wider range of possibilities as to where they were able to work geographically, and in terms of advancing professionally.

While the MD-PhDs used the PhD-degree to position themselves in the medical field, some also presented themselves as being positioned in a larger field by the ward or the hospital they worked at, and as having become part of a prestige project in a time of tight financial control at hospitals and society as a whole. A chief surgeon remarked:

*A ward profiles itself outwardly by having something take place, by being innovative, by doing research, and that’s what everybody’s thinking: “Oh, well that might prompt even more PhDs, and then there will be a flourishing environment and it will become a fabled ward”. But that’s what people want and that’s what the head of departments want. That’s what the board of directors of the hospital wants. They want this [because] this is a time when they are discussing what hospitals are going to remain, and what wards are going to remain, and all wards are under constant pressure.*

MD-PhD, surgical speciality, regional hospital

In this sense, a PhD-degree was not only seen as a form of academic capital for the MD-PhDs themselves, i.e. a form of cultural capital that is gained through the acquisition of an academic qualification, but was subject to the significance it holds outside the educational institution [36]. As shown in the excerpt, some MD-PhDs constructed themselves as constituting a form of academic capital, i.e. as being part of a larger game plan of survival whereby each ward or hospital is positioning itself outwardly as an academically successful environment within the context of hospital closures.

#### The PhD-degree is a way to improve evidence-based clinical practice

When asked directly, none of the interviewed MD-PhDs indicated that they got other assignments than their colleagues without PhD-degrees, although some mentioned that they did more teaching and coaching. However, many expressed that their approach to clinical practice was different than that of their colleagues and than before they did the PhD. They conveyed that the primary quality they had brought with them was assessing and being critical in two respects: In terms of scientific literature, and in the clinic.

##### Assessing scientific literature

The use of scientific literature has become an increasingly bigger part and even a demand of clinical life over the last years in Denmark as in other Western countries. In particular, more and more focus has been put on the use of evidence-based medicine, which has been defined as a systemic approach to analyze published research as the basis of clinical decision-making [37]. The interviewed MD-PhDs argued that their PhD-education helped them in terms of being able to form an overview of the constantly incoming new scientific literature. Some of the MD-PhDs expressed in a straight forward manner that the PhD-degree helped them carry out evidence-based medicine in the clinic. Thereby they were able to perform a form of translational medicine, i.e. bridging the gap between science and the clinic by “translating” the newest scientific knowledge into application in the clinic.

Furthermore, the MD-PhDs conveyed that the PhD-education had equipped them with the ability to critically assess the results of scientific research, especially in terms of the quality of studies but also in terms of their applicability to their own patients. A staff specialist expressed it in this way:

*The medical study is not the place for learning a critical approach. Not at all. And that’s what I think I’ve learned. That the most important thing I’ve gained […] being source critical and critical on the whole.*

MD-PhD, medical specialty, regional hospital

##### Assessing the clinic

Some of the interviewed MD-PhDs expressed concern that that they had “*fallen behind in the clinic*”, i.e. that they were less clinically experienced than their peers, because they had taken 3 years out to do their PhD. However most argued that their clinical skills had improved in other ways. First of all, several MD-PhDs in various ways expressed how they had become more critical towards clinical practice, which in their own perspective made them more likely to look for alternative and better treatments for their patients and not settling for the easy or traditional solution. A chief surgeon expressed it this way:

*I think you become a better clinician by being a researcher [Interviewer: How so?] The approach you have to the problems you encounter that you can’t just manage or if you’ve had a patient who has something you didn’t really know about, I think you’re much quicker to […] make a literature search on the latest evidence in this area, what’s the approach, is any new research being done? […] Is this something we should bring up? How is this managed in other places? I think you’re a lot quicker in doing that if you have a research related background than if you don’t have a research related background. Then you may be quicker to just say “Oh well, that’s that then”.*

MD-PhD, surgical specialty, university hospital

Having a PhD also seemed to position the MD-PhDs as someone who were entitled to speak up and voice their opinion, even if they disagreed with their superiors. In positioning theory, this is referred to as footing, i.e. feeling you are in a position where your words have weight and your opinion matters. A chief physician said:

*Before [the PhD-degree] when an older, more experienced colleague said “Then you do this and then you do that”, I might have been more inclined to just swallow it, and now I’m more “But why? What is really... Could it be done in a different way?” Or when you start something: “Are we certain that this is the right way?”. I think you become more critical towards the treatment.*

MD-PhD, medical specialty, regional hospital

In this sense, the PhD-degree seemed to change the footing in the wards by putting the MD-PhDs in a position where they could speak up, because they had the ability to refer to evidence-based medicine and scientific results.

#### The PhD-degree entails a duty to do research

As described above, the MD-PhDs generally argued that the PhD-education had made them better clinicians in different ways. Yet as MD-PhDs they seemed well aware of their double position as both clinicians and as scientific researchers, but there was great difference as to how much of their time they spent doing research. A few of the interviewed MD-PhDs conveyed being very research active in terms of publishing peer reviewed articles and seeking foundation grants, while most stated that they did not spend a lot of time doing research because of busy work schedules in the clinic. However, most stated that they expected to do research in the future.

There were also great differences as to how many working hours the MD-PhDs expressed they were allotted to do research. One MD-PhD had one or two research days a week, while two others had 1 day (an “*office day*”) to do research a month, while most – if they wanted to do research – had to do so on their own time.

When asked what he had obtained by doing a PhD, one MD-PhD answered “*A guilty conscience*” which denoted how problematic it was for him to find time to do research after returning to work in the clinic. The same medical doctor explained:

*[Interviewer: So the research is done on your own time?] Yes. It may be months between working on the protocol. I also have another project that’s still at the idea stage, where I’ve been trying to arrange a meeting between two professors who are both very positive [about the idea], but we simply haven’t been able to coordinate our calendars. Last time we met, we agreed that it might be better for me to take a leave of absence from my position, so I could have some consecutive time, a month or two to finish it. So I’m applying for funding for that now, but it’s a slow process. The idea is at least a year and a half old.*

MD-PhD, medical doctor, regional hospital

Interestingly, when asked about their research activity, MD-PhDs responded that they did no or only very limited research in terms of doing peer reviewed articles. However, later in the interviews they often conveyed that they were in the process of e.g. developing or improving (evidence-based) clinical guidelines or doing small scale, local studies that were not published internationally. This indicates that other types of research do not count as research in the same way as peer reviewed articles.

Some of the MD-PhDs expressed frustration that they did not do more research and that they were not allotted time to do so in their work schedule, because they found it hard to balance their working and personal lives and still find time to do research in their off hours. A few, however, voiced the opinion that to them it seemed quite acceptable that research had to take place in one’s spare time. A chief surgeon said:

*You have to have some drive. You can’t just be a wage earner in this field […] Those who contribute something extra, it is fair that they are prioritized and given something extra in other contexts. You should be honored for the amount of work you put into it. Not financially, but professionally.*

MD-PhD, surgical specialty, university hospital

Even though they expressed frustration that they lacked of time to do research, everyone concurrently expressed that they found it important to do research in the clinic. Many emphasized that it was in the clinic that ideas for research emerged, because it was in the clinic one observed and became curious about different problematic issues. In this way, they position themselves as someone who takes on the double role of being a clinician and being a researcher at the same time. A chief physician said:

*The place where you get the ideas for a lot of things [research], is when you’re out where the patients are. “Here we might do things like this”. […]And if you are going to have an idea about how to do things differently, you have to have experienced the problem yourself and seen “We have a problem here. We must be able to do things in a different way”. And that’s the innovative thing you learn from being a PhD, which signifies that we need clinicians to have a research education as well.*

MD-PhD, medical doctor, university hospital

Because of time pressure, rather than doing research themselves, some of the MD-PhDs conveyed that they contacted other (younger) physicians, when they identified a problem in the clinic, they felt warranted further examination. A chief physician said:

*I’ve started changing my thinking so that I’m not the one who has to do everything, because then we’ll get nowhere, but it’s more about finding something [a problem] and then knowing what the research procedure is, how to get a protocol started and get the funding and then serve it more as a package, so it’s easier to get younger colleagues [to do it].*

MD-PhD, medical doctor, university hospital

### Colleagues’ storylines

#### MD-PhDs are resource persons

Five of the seven physicians without a PhD-degree we interviewed reflected the MD-PhDs’ own understanding of MD-PhDs as helping their (especially younger) colleagues in the clinic. Colleagues expressed that MD-PhDs primarily helped them in two ways. First in terms of helping them doing or starting their own research projects. The five physicians conveyed that their MD-PhD colleagues were extremely helpful in terms of helping them write protocols, make posters, seek funding, etc. In this way, the physicians positioned the MD-PhDs as resource persons who contributed with knowledge and guidance. One physician said:

*They do a lot of the teaching because of their [research] education, but most of all they also use their education to, like, help others get started doing research. [At this hospital] there’s a lot of young people, and you don’t get far today, if you don’t do some kind of research. That’s where you need someone a little older to support you.*

Medical doctor, regional hospital

Secondly, the colleagues also positioned the the MD-PhDs as someone who increased the focus on science and the use of evidence-based research in the clinic. One physician said:

*I think they’re really good at [helping] if there is something I’m unsure about or [if I] have some questions at the care conference. They’re really good at reading articles and looking at reviews and stuff like that. I think they have a more academic approach than for instance I do. […] I think they are really good at contributing and also starting some things in terms of new instructions and calling into question some of the things the older [physicians] say, about “That’s the way we do it, because that’s the way we’ve always done it”. […] They’re quicker in terms of seeing through advantages and disadvantages of different studies, and I think I find that difficult sometimes, when I read literature. It’s not like I don’t read any literature, but I’m more a person who reads reviews because I find it difficult to see through the original articles.*

Medical doctor, regional hospital

The above quote also shows that the physicians positioned the MD-PhDs as someone with footing, i.e. they are able to challenge their older, more experienced colleagues by referring to evidence-based research, and thereby shifting the focus from clinical tradition to evidence-based practice.

#### MD-PhDs hold an unjustly favored position

On the other hand, rather than accentuating the footing of the MD-PhDs, two of the seven interviewed physicians without PhD challenged the footing of the MD-PhDs. They did so in different ways. First, they distinguished between those MD-PhDs who were research active and those who were not. These two physicians clearly expressed that they saw it as a duty of the MD-PhDs to be research active. One of them said:

*One might have expected them [MD-PhDs] to be research active. That they continued some projects, but that is probably more moderate. Once you have your PhD you go… Maybe it’s natural once you’ve spent a lot of time doing something, and then when you’re done, maybe you need a break. Then there’s the risk that you get out of your usual course.*

Surgical doctor, university hospital

Here active research is seen as what should distinguish the MD-PhDs from other physician positions, yet this duty is seen to be neglected. In the above quote, the very polite physician seems to be finding excuses for the MD-PhDs (“*maybe you need a break*”), yet in the interview situation it was clear that he believed that they were under obligation to put their (expensive) education to better use by doing some kind of research.

Furthermore, the two physicians not only faulted (many of) the MD-PhDs for not doing research, but also challenged their academic capital [33]. Both conveyed that in their opinion the MD-PhDs had taken out a patent on the scientific approach, which left the clinicians to be viewed as less scientific in their approach. A chief physician said:

*I think it’s an important point that there is this rhetoric that has to do with […] if you decide to take the PhD-track, then you learn something about scientific thinking. You’re someone who’s inclined to search for an article […]. If you have a clinical question, you don’t just stand around thinking “Hmm, should I ask someone I know?” No, you seek new knowledge yourself. Yeah, you know what? You can do that even if you don’t have a PhD. You can easily be scientifically well-founded without three years of something. What kind of nonsense is that? Off course you can be a researcher and evidence-based without having [a PhD-degree].*

Medical doctor, university hospital

Thus, physicians without a PhD-degree may find that MD-PhDs are put in an unjustly favored position. The physician from the above quote talked about how it often goes unrecognized that the competencies and skills of MD-PhDs can also be achieved in other ways and conveyed that physicians who had not done a PhD “*sort of have to qualify themselves additionally*”. He stated:

*There’s a prestige about writing a PhD that I sometimes find hard to understand […] [T]hen they write “PhD” at the bottom of their e-mail and then suddenly that means that […] there are things we no longer need to discuss, because now they’ve sort of qualified themselves. […] And I find myself thinking sometimes: “Well, it’s not like I’ve just been asleep in a chair while you wrote your PhD, right?”*

Medical doctor, university hospital

### Leaders’ storylines

In the following, the term *leaders* is used to denominate executive consultants and members of appointment committees.

Overall, the interviewed leaders conveyed that they did not believe they gave MD-PhDs other or different tasks than physicians without PhD. However, the leaders also expressed different expectations of MD-PhDs and primarily positioned them in three different ways, as will be described below.

#### MD-PhDs are resource persons

In general, the leaders expressed that they did not find it relevant what subject the MD-PhDs had done their thesis on. Instead they stressed the generic skills the MD-PhDs had achieved by doing it. Approximately half of the leaders conveyed that they primarily regarded the PhD program as an outright “*learning process*” and a “*basic schooling*” in regards to academic skills, in particular in terms developing a critical approach to use of research literature and to the clinic. Some said that in their opinion, critical thinking was not a skill that was taught sufficiently in medical school, as the following quote exemplifies:

*Medical students don’t go to university, they go to “*doctor school*” and come out completely identical like a little machine. When you do a PhD, you learn to think independent thoughts.*

Member of appointment committee

Some members of appointment committees independently described how the medical profession in their experience had become exceedingly characterized by a rapidly growing amount of new research results constantly coming in, and that it was crucial that physicians in the clinic are able to not only follow but also reflectively assess research results that come in. Some underlined that this enabled the MD-PhDs to work in a highly evidence-based way, but also that it made them able to critically evaluate research results and their applicability in the clinic. A member of an appointment committee said:

*It’s important to be critical towards science and know about basic statistics, because a lot of treatment and innovation requires that you are able to be critical of what you read, and that’s not something that you’re born with. That’s something you learn.*

Member of appointment committee

Thus, the leaders described that the MD-PhDs were experienced at making use of their strengthened skills as scholars, which is one of the seven roles of physicians. Originally developed in Canada, the model “The seven roles of physicians” is used in Denmark to describe the competencies required of medical specialists [38]. A number of the interviewed leaders stated that some of the other seven roles of physicians improved for MD-PhDs as well. These leaders emphasized that in their experience MD-PhDs were also strengthened in the roles of collaborator, communicator and managers/administrators, since making a PhD will almost inevitably involve e.g. collaborating with a wide range of people, communicating results, and keeping to one’s schedule.

Some leaders mentioned a range of other competencies and skills which MD-PhDs in their experience also had or obtained, e.g. working in a structured way, overcoming obstacles, and being curious and committed, ambitious and goal-oriented, as well as working independently.

Furthermore, this group of leaders conveyed that the MD-PhDs’ evidence-based and scientific approach to the clinic influenced and inspired younger physicians, creating what an executive manager termed “*a research culture*”. A member of an appointment committee stated:

*They* [MD-PhDs] *are engines for being curious, for being initiators, they are water carriers in terms of getting those who have just come from an introductory position and don’t have a scientific background into some academic communities and showing an academic way of thinking to the younger physicians and in that sense becoming role models in this area. They are part of improving the standard and the drive among younger physicians.*

Member of appointment committee

#### The PhD-degree entails a duty to do research

While some of the leaders, as just described, positioned MD-PhDs as having particular skills that raised the standard of clinical treatment, another portion of the interviewed leaders distinguished quite strictly between those MD-PhDs who were research active and those who were not. A member of an appointment committee said:

*I think you can almost put them in two boxes, these PhD-people. There are some who do a PhD and then it ends there, because then we’ve sort of done what we could for the career, they think. That’s a bad PhD. And then there are some who do a PhD, because they find it interesting and continue to initiate things.*

Member of appointment committee

The leaders expressed a need for MD-PhDs to do research, not only to contribute to medical progress, but also because they contribute to the clinical development and do research in the ward that is implemented with the patients. This, in turn, was seen as strengthening the “*research culture*” of the wards. A member of an appointment committee explained:

*If you don’t have any research in a ward, then [..] people can’t construe the treatment methods that come in. And the interest in doing new things will probably disappear as well, if you don’t have a research environment. Then no one is inspired to try new things. Not as easily anyway. […] If no one is interested in research, then no one wants to read the national guidelines, and then they are not followed. And you can see that in many wards, that that’s how it is. And I think it’s of great importance that we follow them. That people know them and that we follow them. That’s the kind of thing that can be derived from research, right. And then it’s inspiring.*

Member of appointment committee

#### MD-PhDs hold an unjustly favored position

The leaders who distinguished between whether MD-PhDs were research active or not also challenged the distinctiveness of MD-PhDs in two ways. First, some pointed out that in their experience you might *expect* MD-PhDs to have achieved all of the aforementioned skills (critical assessment of literature, etc), but that did not mean that they had in fact achieved them. Furthermore, some pointed out that those were skills that were expected of all physicians. A member of an appointment committee conveyed:

*Fundamentally, if you have a medical specialist in a subject area, then that person – PhD or not – has to be able to assess, has to be able to dive into specialized knowledge about demarcated subjects, if a patient shows up, where that is required.*

Member of appointment committee

Secondly, some claimed that the qualities some achieved by doing a PhD could also be achieved in different ways. A member of an appointment committee said:

*I also find it important to point out that this palette of qualities you can achieve as a PhD are also qualities you can get in all other kinds of ways […] via different activities and via some work on the side. […] It takes some basic things to complete a PhD, but there are also other ways around.*

Member of appointment committee

## Discussion

The aim of this article has been to explore the positioning of MD-PhDs in the clinic by means of qualitative interviews with a number of MD-PhDs and their leaders and colleagues (physicians without PhD) with a specific reference to the positioning of the MD-PhD.

Our analysis using positioning theory showed that the interviewed MD-PhDs positioned themselves as someone who primarily used their research competencies in the clinic and only to a lesser extent by doing research themselves. The interviewed colleagues mainly positioned their MD-PhD colleagues in two different ways. First, as resource persons in terms of assisting in doing scientific research and by increasing the focus on research and evidence-based medicine in the clinic. Secondly, some positioned the MD-PhDs as underserving of what they regarded as the MD-PhDs’ high status, since they were not (all) research active and because they were put in an unjustly favored position. Finally, the leaders could be divided into two approximately equally sized camps who disagreed as to the positioning of the MD-PhDs. While one side described the PhD-degree as a “basic education”, thereby positioning MD-PhDs as resource persons in the clinic, the other side insisted that the PhD entails a duty to do research and that research is the MD-PhD’s raison d’etre since the basic skills hailed by others were regarded as achievable in other, less expensive ways.

As such there was great diversity as to whether the interviewed physicians as a whole found the MD-PhDs to be living up to their duties, and as such there were two dominant story lines. While one story line focused on ability of the MD-PhDs to assess and implement research in the clinic, the other storyline focused on their research activity (or lack thereof) and distinguished between those MD-PhDs who were research active and those who were not. The main difference in the positioning of MD-PhDs thus revolved around whether doing research or whether using other competencies obtained during the PhD-education were sufficient to fulfill one’s duties. As such the positioning, i.e. the tasks and duties, of MD-PhDs came across as less than clear-cut in the clinic in this study.

As with most qualitative studies, a relatively small sample size limits the study’s generalizability. Furthermore, we used purposive sampling, which is a non-representative method of recruiting, to recruit physicians for the study. Therefore it is possible that we did not get in contact with physicians who e.g. were uninterested in science in the clinic and/or were indifferent to MD-PhDs. Moreover, the empirical data that the study is based on stems mainly from Central Denmark Region and from selected medical and surgical specialties. Attitudes and experiences might be different in other Danish regions and in other specialties.

In many ways, our empirical findings mirror the public and professional debate in Denmark, which revolves around whether or not too many MD-PhDs are being trained. Based on our relatively small empirical study, we are unable to assess whether more or less MD-PhDs are warranted. Instead our findings call for a discussion of how best to make use of the dual-degree, as will be elaborated on below.

In our empirical study about how MD-PhDs contribute to the everyday clinical setting, there are two opposing positions with one side critiquing the MD-PhDs for not doing enough research and for merely using the PhD-degree to climb the career ladder, while the other side emphasizes the MD-PhDs’ potential for integrating medical scientific knowledge with clinical decision making and thereby enhancing patient care. Put in others terms, it seems the former position blames the MD-PhDs for not doing enough *bedside to bench* research, while the latter praises them for doing *bench to bedside* research or so-called translational medicine.

Although the situation in Denmark is different from the situation internationally, there are also similarities. Recently a number of countries have been concerned that the number of MD-PhDs/physician scientists is decreasing [6–9], especially in the USA, where the physician scientist pipeline has been characterized as challenged [39]. However, some publications point out that there seems to be general confusion as to what the MD-PhD/physician-scientist position should encompass which is consistent with the situation in Denmark. Recent studies point out that there are no formal standards as to what is expected of the MD-PhDs, and how their different competencies are best utilized [2, 4]. Even in the Danish Ministerial Order on the PhD Programme, there seems to be an openness to interpretation as the order by on the one hand emphasizing that “*[t]he PhD programme is a research programme aiming to train PhD students at an international level to undertake research*” [40], but also in an additional note declares that that the education not only aims to educate to do scientific research but equally to “*broader functions in the private and public sectors*” [41]. [Our translation]).

Such equivocality, in turn, makes it difficult to measure the MD-PhDs’ value. Often they are judged by their number of publications [18], but as we found and as pointed out in other studies, the MD-PhDs also contribute in other - less measurable – ways. For instance, Garrison & Deschamps [42] emphasize that MD-PhDs and physician scientists make valuable contributions to research in other ways than as a principal investigator by serving as consultants and advisors or collaborating on grants. In our study, we found that the MD-PhDs contribute in terms of teaching, enhancing the focus on evidence-based medicine in the clinic and doing translational medicine as well as doing local research studies and developing clinical guidelines.

Sutton & Killian [3] describe how the purpose of dual-degree training in the biomedical sciences has also been broadly interpreted in the USA and that the types of careers MD-PhDs can be expected to pursue subsequently have been a point of some confusion. While some regard MD-PhD programs as “*a flexible approach to scientific training producing both basic scientists and clinical investigators*”, others “*tend to view these programs as generating either one type of researcher or the other*” [3] (p. 454). Sutton & Killian argue that the diverse range of expectations associated with dual-degree programs has “*complicated the efforts of planners and policy makers in projecting workforce needs and generating recommendations for research training*” (Ibid.). In the Danish setting, there seems to be a similar kind of confusion in terms of the ways in which the MD-PhDs are expected to contribute in the clinic, although the dispute here has to do with whether the MD-PhDs first and foremost are researchers who also work in the clinic or whether they are scientifically upskilled clinicians. The central question, according to Sutton & Killian, is whether it has to be a choice, i.e. whether MD-PhDs must either pursue research (and rarely see patients in the clinic) or practice medicine (and have nominal research activity) or whether it is possible to have a career path that can unify the two (Ibid.).

Our findings show that the lack of consensus as to the rights and duties of MD-PhDs may lead to frustration in leaders and colleagues who feel that the MD-PhDs are not living up to their responsibilities in terms of doing research. However, the lack of consensus also seems to cause frustration for the MD-PhDs themselves, putting them in an unresolved or ambiguous position where they are judged as both clinicians and researchers despite great variation as to how many working hours are allotted their research; many having to do research in their off-hours. A clarification of what is expected of MD-PhDs in the clinic might alleviate some of this frustration as well as sparking a debate about how MD-PhDs can best spend their work day.

In a similar vein and with an individual focus, Rosenblum et al. [43] have recently tried to define the nature of clinician-scientist professional identity to understand the underlying motivations and actions that underlie the decision to enter this career track and to remain a clinician-scientist, claiming that understanding the challenges of being a clinician-scientist might contribute to greater career sustainability. With a similar focus on the individual level, it has been suggested that physician scientists need special encouragement and tools in order to balance clinical and research duties as well as balancing (personal) life and career [44].

We want to propose the idea that the individual difficulties as well as the discrepancies found in the public and scientific debate and in our empirical study might also be symptomatic of a more general question which has to do with the place science holds in the clinic. Medicine has been described as an uneasy juncture of science and art [45], and especially within medical academia, there is an ongoing debate as to whether medical practice is primarily an art (in the sense of being a skill acquired by experience and not an exact science) or a science (relying on research and scientific evidence to shape guidelines for clinical outcomes) [46]. The same debate seems to be evident in the way some point out that MD-PhDs lack clinical experience and skills compared with their MD-only colleagues while at the same time clinical knowledge and wisdom is often perceived as secondary to scientific knowledge [47]. As a practical solution to this dilemma, a closer integration of clinical and research training, i.e. the combination of clinical care and scientific research, has been proposed [48, 49].

Thus, although different approaches have been taken pertaining to clarifying and resolving the position of science (and MD-PhDs) in the clinic, including what counts as scientific research, it seems safe to observe that more research, clarification and deliberation is needed in this area.

## Conclusions

Our study underlines that in Denmark the positioning of MD-PhDs in the clinic is diverse and equivocal, because some, broadly speaking, focus on the MD-PhDs’ ability to translate and implement research in the clinic, while others insist that MD-PhDs need to be research active. Although the situation in Denmark differs from that of a range of other countries, because the debate here revolves around whether too many MD-PhDs are being educated rather than how to attract more physicians to the dual-degree, there seems to be a general dispute as to how the success of MD-PhDs is defined and measured. By establishing more clearly how we wish to position physicians with a dual-degree in the clinic, we might get a better idea of how many to educate and how to make better use of their competencies. With this study we wish to draw attention to the circumstance that a conceptual debate about the expectations of the MD-PhDs is warranted.

## References

[1] Donovitz M, Germino G, Cominelli F, Anderson JM (2007). The attrition of young physician-scientists: problems and potential solutions. Gastroenterology.

[2] Marsh JD, Todd RF (2015). Training and sustaining physician-scientists: what is success?. Am J Med.

[3] Sutton J, Killian CD (1996). The MD-PhD researcher: what species of investigator?. Acad Med.

[4] Brass LF, Akabas MH, Burnley LD, Engman DM, Wiley CA, Andersen OL (2010). Are MD–PhD programs meeting their goals? An analysis of career choices made by graduates of 24 MD–PhD programs. Acad Med.

[5] Cox TM, Brimicombe J, Wood DF, Peters DK (2012). The Cambridge Bachelor of Medicine (MB)/Doctor of Philosophy (PhD): graduate outcomes of the first MB/PhD programme in the UK. Clin Med.

[6] Koike S, Ide H, Kodama T, Matsumoto S, Yasunaga H, Imamura T (2012). Physician–scientists in Japan: attrition, retention, and implications for the future. Acad Med.

[7] Lander B, Hanley GE, Atkinson-Grosjean J. Clinician-scientists in Canada: barriers to career entry and progress. PLoS One. 2010;5(10) 10.1371/journal.pone.0013168. 10.1371/journal.pone.0013168 PMC2949393 20957175

[8] Milewicz DM, Lorenz RG, Dermody TS, Brass LF, the National Association of MD-PhD Programs Executive Committee (2015). Rescuing the physician-scientist workforce: the time for action is now. J Clin Invest.

[9] Schölmerich J (2010). Where have all the physician scientists gone. Ger Res.

[10] Wyngaarden JB (1979). The clinical investigator as an endangered species. N Engl J Med.

[11] Rosenberg LE (1999). Physician scientists: endangered and essential. Science.

[12] Schafer A (2009). The vanishing physician-scientist?.

[13] Schwartz DA (2012). Physician-scientists: the bridge between medicine and science. Am J Respir Crit Care Med.

[14] Universities Denmark. Satsningen på ph.d.-uddannelse [the focus on PhD-education]. Denmark: Universities Denmark. 2013. https://dkuni.dk/wp-content/uploads/2017/10/ph-d-publikation-170113-p.pdf. Accessed 19 Feb 2018.

[15] Andreassen P, Wogensen L, Christensen MK. The employers’ perspective on how PhD training affects physicians’ performance in the clinic. Dan Med J. 2017;64(2):1603–9629. 28157064

[16] Jeffe DB, Andriole DA, Wathington HD, Tai RH (2014). The emerging physician-scientist workforce: demographic, experiental, and attitudinal predictors of MD PhD program enrollment. Acad Med.

[17] Uddannelses & forskningsministeriet [Ministry of higher education & science in Denmark]. Phd-uddannelsens kvalitet og relevans: Sammen skrivning af hovedresultater [The quality and relevanse of the PhD education: Main results]. Accessed 19 Feb 2018 from https://ufm.dk/publikationer/2017/filer/ph-d-uddannelsens-kvalitet-og-relevans.pdf.

[18] Fosbøl EL, Fosbøl PL, Rerup S, Østergaard L, Ahmed MH, Butt J, Davidsen J, Shanmuganathan N, Juul S, Lewinter C (2016). Low immediate scientific yield of the PhD among medical doctors. BMC Med Educ.

[19] Skajaa K, Djurhuus JC. Er ph.d.-læger gode læger? [Are MD-PhDs good doctors?]. Ugeskr Læger [Wkly J Doc]. 2016;7:0041–5782.

[20] Skipper M (2013). En ph.d. skal kunne bruges [A PhD should be of use]. Ugeskr Læger [Wkly J Doc].

[21] Aarhus University (2015). Beskæftigelsesundersøgelse 2014: Rapport for ph.d.-dimittender [Employment study 2014: Report for PhD graduates].

[22] Patton MQ (2002). Qualitative research and evaluation methods.

[23] Kvale S, Brinkmann S (2009). InterViews: learning the craft of qualitative research interviewing.

[24] Bernard HR (2006). Research methods in anthropology: qualitative and quantitative approaches.

[25] American Anthropological Association. Statement on ethics: Principles of professional responsibilities. 2012. http://ethics.americananthro.org/category/statement/. Accessed 19 Feb 2018.

[26] Emerson RM, Fretz RI, Shaw LL (1995). Writing ethnographic fieldnotes.

[27] Moravcsik A. Transparency: The Revolution in Qualitative Research. 2014. http://www.princeton.edu/~amoravcs/library/transparency.pdf. Accessed 19 Feb 2018.

[28] Patton MQ (1999). Enhancing the quality and credibility of qualitative analysis. Health Serv Res.

[29] Langenhove L, Harré R, Harré R, Langenhove L (1998). Introducing positioning theory. Positioning theory: moral contexts of intentional action.

[30] Harré R, Moghaddam FM, Cairnie TP, Rothbart D, Sabat S (2009). Recent advances in positioning theory. Theory Psychol.

[31] Davies B, Harré R (1990). Positioning: the discursive production of selves. J Theory Soc Behav.

[32] Harré R, Moghaddam F, Harré R, Moghaddam F (2003). Introdution: the self and others in traditional psychology and in positioning theory. The self and others: positioning individuals and groups in personal, political, and cultural contexts.

[33] Harré R (2005). Positioning and the discursive construction of categories. Psychopathology.

[34] Harré R, Slocum N (2003). Disputes as complex social events: on the uses of positioning theory. Common Knowl.

[35] Goffman E (1981). Forms of talk.

[36] Bourdieu P (2010). Distinction: a social critique of the judgment of taste.

[37] Claridge JA, Fabian TC (2005). History and development of evidence-based medicine. World J Surg.

[38] Danish Health and Medicines Authority. The seven roles of physicians. 2013. https://www.sst.dk/en/news/2013/~/media/39D3E216BCBF4A9096B286EE44F03691.ashx. Accessed 19 Feb 2018.

[39] Daye D, Patel CB, Ahn J, Nguyen FT (2015). Challenges and opportunities for reinvigorating the physician-scientist pipeline. J Clin Invest.

[40] PhD-bekendgørelsen [Ministerial Order on the PhD Programme]. 2013. http://phd.ku.dk/hoejrebokse/phd-bekendtgoerelse-da/Engelsk_-_ph.d.-bekendtg_relse__2_.pdf. Accessed 19 Feb 2018.

[41] Ministeriet for Forskning, Innovation og Videregående uddannelser. 2013. http://samf.ku.dk/phd-skolen/pdf/Vejledning_ph_d_-bekendtgoerelsen_af_27._august_2013.pdf. Accessed 19 Feb 2018.

[42] Garrison HH, Deschamps AM (2014). NIH research funding and early career physician scientists: continuing challenges in the 21st century. FASEB J.

[43] Rosenblum ND, Kluijtmans M, Olle C (2016). Professional identity formation and the clinician–scientist: a paradigm for a clinical career combining two distinct disciplines. Acad Med.

[44] Morel PA, Ross G (2014). The physician scientist: balancing clinical and research duties. Nat Immunol.

[45] Battista RN, Hodge MJ, Vineis P (1995). Medicine, practice and guidelines: the uneasy juncture of science and art. J Clin Epidemiol.

[46] Balaban CD (2008). Toward revitalizing the role of physician-scientists in academic medicine. Otolaryngol Head Neck Surg.

[47] Panda SC (2006). Medicine: science or art?. Mens Sana Monogr.

[48] Goldberg C, Insel PA (2013). Preparing MD-PhD students for clinical rotations: navigating the interface between PhD and MD training. Acad Med.

[49] Harding CV, Akabas MH, Andersen OS (2017). History and outcomes of 50 years of physician-scientist training in medical scientist training programs. Acad Med.

